# Environmental DNA (eDNA) detects the pool frog (*Pelophylax lessonae*) at times when traditional monitoring methods are insensitive

**DOI:** 10.1038/s41598-018-23740-5

**Published:** 2018-04-03

**Authors:** Alexander Eiler, Anders Löfgren, Olle Hjerne, Sara Nordén, Peter Saetre

**Affiliations:** 1 eDNA solutions AB, Björkåsgatan 16, SE-43131 Mölndal, Sweden; 2 EcoAnalytica, Slalomvägen 28, SE-129 49 Hägersten, Sweden; 3 Svensk Kärnbränslehantering AB, Box 3091, SE-169 03 Solna, Sweden

## Abstract

Detection of endangered species is invaluable for conservation efforts, yet many traditional sampling techniques are ineffective at low population abundances or during certain periods of the year. Here, we compared results from a newly developed eDNA approach and the traditional observational method for the endangered pool frog (*Pelophylax lessonae*). Analysis using an occupancy-modeling framework indicated that the probability of pools being occupied using eDNA (0.93) was higher than for the traditional method of counting calling males and silent observed individuals (0.72). Detailed analysis revealed complementarity among the methods. That is, the traditional method gave a high rate of observation in June, whereas eDNA gave at least as many or more observations during other parts of the year. Discrepancies among the methods depended on the dominant lifecycle stage, and eDNA concentrations were higher when juveniles were present than at times when spawning occurred. eDNA concentrations were also positively related to *P. lessonae* observations. Our study demonstrates that an eDNA protocol for monitoring of endangered amphibian species can be particularly valuable during periods when individuals are hard to detect by observational methods, and provides guidance to sampling efforts for research and monitoring programs in other regions and systems.

## Introduction

Freshwater systems are threatened by biodiversity loss, with freshwater fauna experiencing over 100 documented extinctions in the 20th century^1^. Extinction rates for freshwater animals are estimated to be as high as 4% per decade, five times greater than species losses in terrestrial systems^2^. Factors underlying freshwater biodiversity decline include overexploitation of water and organisms, water pollution, and habitat destruction and degradation, all of which are linked to human activities^2^. Superimposed on these factors are global-scale environmental changes, such as climate warming and acidic deposition. A primary reason for concern over the current accelerated loss of species is the proposed associated loss of ecological functions including ecosystem services such as drinking water and fisheries^2,3^.

However, investigations into the faunal biodiversity are often hindered by the challenges posed by aquatic systems. The difficulty to inventory is due to the complexity of topography and vegetation, water body characteristics, low densities of individuals, cryptic coloration, and the use of microhabitats^4–8^. As a result, surveys for many aquatic species, such as amphibian, can be expensive and inaccurate. Furthermore, traditional methods to obtain species inventories are invasive and selective and can be only carried out in particular areas or at times when conditions are favorable. For example, even though electrofishing techniques are often successful in detecting aquatic vertebrates, they can be time consuming and difficult to apply, and may cause injury to target and non-target species.

DNA from various specimens has been used to detect terrestrial^9–11^ and aquatic^12,13^ vertebrate species for over a decade now, and detection of microbial species using environmental DNA (eDNA) found in soil, freshwater and seawater is revolutionizing species inventories^14–16^. The reliable detection of aquatic vertebrate species using eDNA in water was confirmed in wetlands^17^ and in a large river and canal system^18^. Using eDNA to detect rare and secretive species has been shown to increase accuracy and decrease costs of environmental surveys^19^, to increase the number of sites sampled per unit effort^18^, to refine distribution and extinction records^6^, and to provide early detection of decline in endangered species without any risk for the species^20^.

The pool frog (*Pelophylax lessonae*) lives on the fringe of its global distribution having a long hibernation period and inhabiting small shallow ponds with a short temperature window for reproduction^21^. The population has shown to resemble a metapopulation structure, where small separated populations, corresponding to the regional habitat distribution, are more or less prone to extinction depending on interpatch migration (e.g.^22,23^). This makes them very vulnerable to habitat fragmentation, and forestry pose a severe threat as it obstructs their migration, which might endanger the survival of the entire population. The International Union for Conservation of Nature (IUCN) has the pool frog listed as least concern (LC), the lowest level of threat, internationally [http://www.iucnredlist.org/details/58643/0]. In Sweden however, it is listed as vulnerable (VU) due to the fact that its habitats are declining and exhibit increasing fragmentation [http://www.iucnredlist.org/details/58643/0]. Thus, extensive and modern monitoring strategies are required to assess the consequences of human pressures on conservation efforts of *P. lessonae* and biodiversity overall.

Using eDNA as a monitoring approach for *P. lessonae* was conceived as an alternative to traditional field monitoring approaches, such as counting calling males and silent observed individuals, because of the need to optimize observational effort according to weather conditions (i.e. sunshine, air temperature and wind speed). Also, pool frog activity is different during the season and may be unevenly distributed throughout a site. Differences between methods in the probability of detecting individuals may have large implications on the perceived distribution of a species within an area. Here we investigate if eDNA techniques may provide an efficient and noninvasive mean of detection at low abundance.

## Results

**Figure 1:**
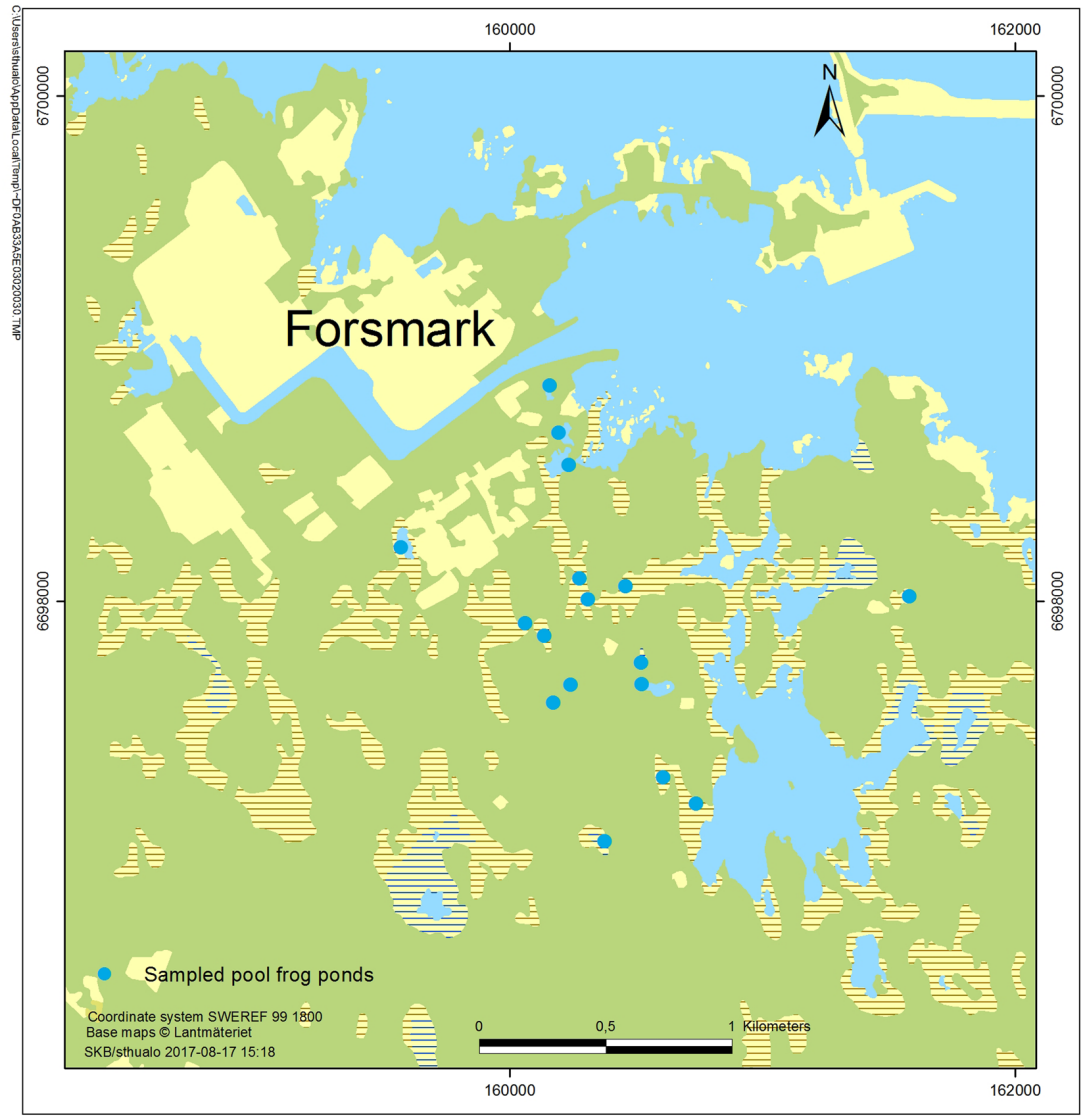
Sampling sites and entrance points of the 17 sampled pools.

In total seventeen ponds in the area located at the Swedish East coast (Baltic Sea) in the eastern part of the County Uppland close to Forsmark (Lat 60° 22′ N, Long 18° 11 E, see Fig. 1) were visited at least twice during spring and summer 2016 to collect water samples for eDNA analysis (see Fig. 1 detailing sampling sites). This sampling campaign was coordinated with ongoing field studies of *P. lessonae* using traditional field monitoring approaches, such as counting calling males and silent observed individuals.

### eDNA method evaluation

**Figure 2:**
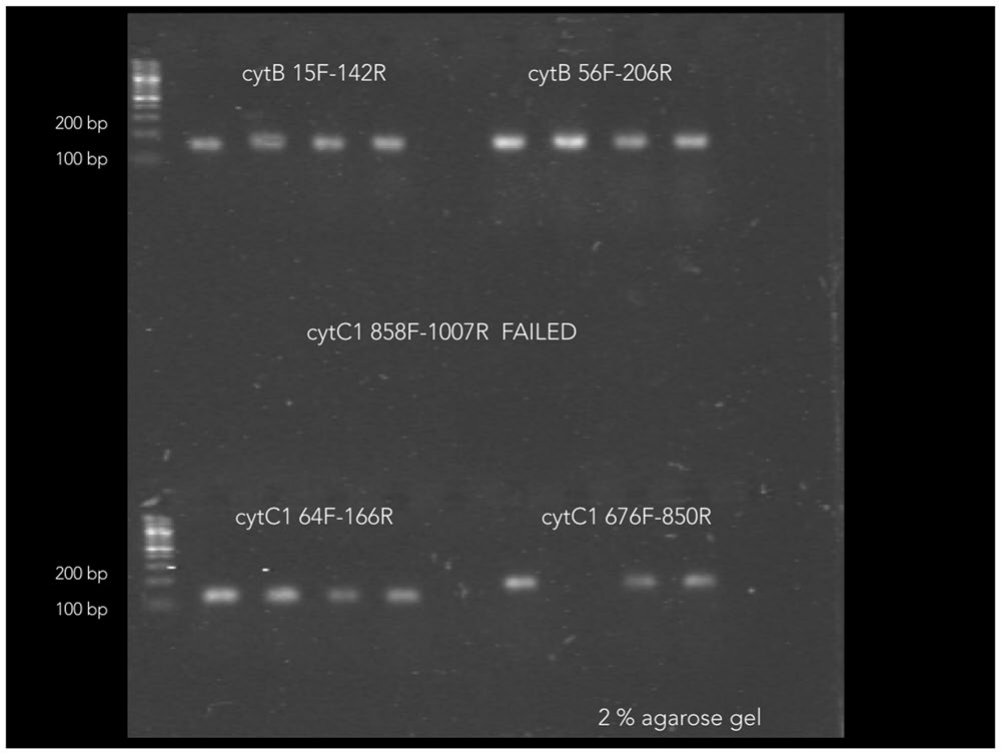
Agarose gel showing products from PCRs using four primer pairs that amplified DNA from a single *P. lessonae* tadpole. As shown in the figure, PCR products ranged in size from 100 to 200 bp, as predicted.

Amplification of *Pelophylax lessonae* with five designed primer pairs (Table 1) was successful, with the exception of primer pair cytC1_858_F/cytC1_1007_R using DNA extracts from a single *P. lessonae* tadpole. The four primer sets that gave positive results (see Fig. 2) were applied to the standard series and an environmental sample spiked with *P. lessonae* tadpole DNA. Results were evaluated based on statistics given by standards and melting curves. Melting curves suggested unspecific amplification in environmental samples when using cytB_15_F/cytB_142_R. R-square (>0.98) and efficiency (>80%) of the qPCR protocols were best for primer pairs *cytC1_676_F/cytC1_850_R* when compared to to the two remaining primer pairs. Thus we chose primer pair *cytC1_676_F/cytC1_850_R* for further analyses using all 67 samples. Using *cytC1_676_F/cytC1_850_R* an additional two replicated QPCR runs including all 67 samples were performed providing triplicated amplification results from each sample.

**Table 1:** *In silico* designed primers for *Pelophylax lessonae* tested in this study.

Primer ID	Sequence		Position	Annealing
cytB_15_F^#^	ATCGCCCAAATCGCAACAGG	forward	15	59.3
cytB_142_R^#^	GAAGAAGGATGCGCCGTTGG	reverse	142	61.3
cytB_56_F^#^	CACAGCTGACACATCCCTTG	forward	50	59.2
cytB_206_R^#^	GCCGTAATATAGGCCTCGTC	reverse	206	59.2
cytC1_64_F*	GGTGCATGAGCCGGGATAGT	forward	64	61.3
cytC1_166_R*	AAGGCGTGGGCGGTAACAAT	reverse	166	59.2
cytC1_858_F*	ATGGGCTCATCACATGTTCA	forward	858	59.2
cytC1_1007_R*	GGGGCTTCCCATTTAATGAT	reverse	1007	59.2
cytC1_676_F*	GACCCCGTTCTCTACCAACA	forward	676	59.2
cytC1_850_R*	ATCCCAGAAGGCCGATAGAT	reverse	850	57.2

^#^Primers for the mitochondrial DNA (mtDNA) cytochrome b (database entry Q94RW7_PELLE).

*Primers for cytochrome c1 (database entry G8HSZ6_PELLE).

Standard curves from each run were statistically evaluated and melting curves were manually checked for unspecific amplification. Target DNA was not detected in any of the negative controls including trip blanks (four samples taken during the winter season), site blanks (pools without *P. lessonae*), filter blanks, extraction blanks and qPCR blanks. Ideally the efficiency (E or slope) of a PCR should be 100%, meaning that for each cycle the amount of product doubles (E = 2), however in our case efficiency was around 80%. R-squares, which are the coefficient of correlation obtained for the standard curves, were above 0.985 in the various PCR runs.

In addition we tested qPCR inhibition by the natural sampling matrix. We spiked three natural samples with a four-fold dilution series of *P. lessonae* tadpole DNA and compared the resulting Cq values with results from the same dilution series in milliQ. qPCR inhibition in the natural samples spiked with *P. lessonae* tadpole DNA was negligible, since qPCRs yielded a Cq diverging from amplification of the dilution series in milliQ by 1.7% (range 0.6 to 3.4%).

### eDNA detection of *P. lessonae*

**Figure 3:**
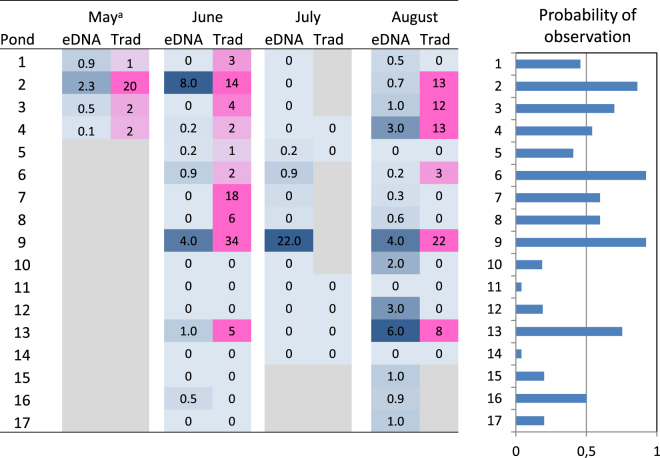
Observations of *P. lessonae* in 17 ponds in Forsmark between mid-May and the end of August 2016. Left) Heatmap showing eDNA concentration in pg per litre (blue) and the number of frogs observed with traditional methods (pink). Right) Between-pond variation in the predicted probability of observing frogs estimated from a joint logistic regression of the two methods. (Bars represent the average of both methods in June). “Months” corresponds to the life-cycle as follows: spawning season (May-June), tadpoles (July) and small and adult frogs (August). a = mean value from three separate visits.

In total, eDNA from *P. lessonae* was observed in 34 samples (57% of 60 eDNA samples) which were obtained from 15 ponds (Fig. 3). The average rate of observation from traditional methods was somewhat lower (47%), and *P. lessonae* was observed at 23 occasions (out of 49) in 10 ponds. There was an overall correlation between the two methods (p = 0.005) and the methods agreed in 69% of the occasions with matched observations (n = 49). In the cases where the method disagreed, eDNA was detected at 9 occasions, whereas traditional methods detected frogs at 6 occasions. Moreover, the number of observed *P. lessonae* was significantly associated to the concentration of eDNA in the sampled water (p = 0.015). However, this association was relatively weak, and further analyses were carried out on observations at the level of presence or absence.

The occupancy analysis was performed to evaluate method-specific detection probabilities, using detection data from 60 sampling replicates across 17 sites (trip blanks were removed). Detection probability for *P. lessonae* was 0.38 per sample with the eDNA method compared to 0.40 when detecting calling males and silent observed individuals assuming that occupancy did not change at a pond within the overall sampling period. These detection probabilities for *P. lessonae* could be translated into a detection probability of 0.93 ± 0.09 compared to a detection probability of 0.72 ± 0.15 of pools being occupied by *P. lessonae* when using eDNA and detection by calling males and silent observed individuals, respectively.

**Figure 4:**
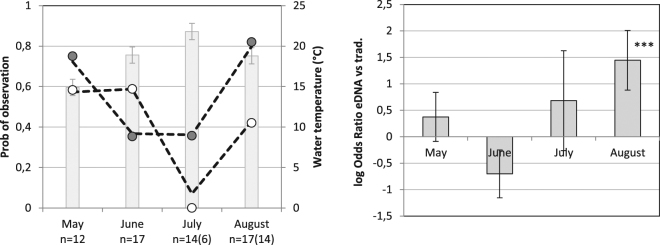
Comparison of methods to detect *P. lessonae* over the sampling period from mid-May to end of August 2016. Left) observed and predicted (dashed line) rate of observation with eDNA (grey circle) and traditional methods (white circle). Grey bar (±S.E.) represent the average water temperature. n = number of samples (observational method in parenthesis). Right) Log odds ratios (±S.E.) for observation with the eDNA vs. the traditional methods. ***Indicate a p-value < 0.001.

As revealed by likelihood ratio tests, the observation rate was depended on both the method of observation (p = 0.009) and time period (p = 0.0008). However, as the effect of the method is strongly depended on time period (p = 0.001), overall comparisons between methods are of limited relevance. Thus comparing methods within time period, the eDNA method had a significantly higher rate of observation in August than the traditional method: the odds ratio (OR) for this time period was 4.2 (p < 0.001) (Fig. 4). The traditional method tended to have a higher rate of observation in June than the eDNA method (OR = 2.0, p = 0.06), whereas the differences between methods were relatively small in May and July as compared to the variation (p > 0.30, Fig. 4).

Furthermore, the likelihood ratio tests revealed that eDNA observation rate was considerably higher in May and August, as compared to the rate in June and July (Fig. 4). Accounting for the effect of which ponds were sampled at the different occasions, the odds of detecting *P. lessonae* DNA during June and July was 2.6 (p = 0.048) and 3.5 (p = 0.014) times lower than in May, whereas there was no significant difference in *P. lessonae* DNA detection odds between May and August. The lowest rate of observation was noted in July, when eDNA was detected in three out of 18 sampled ponds, and no frogs were detected by traditional methods. This highlights the difficulties of detecting *P. lessonae* in a period when tadpoles are expected to be present.

Possible causes of the variation between time periods (within pond) in the *P. lessonae* DNA detection was examined by relating the observation rate to water temperature and filtering conditions. The rate of eDNA observation decreased with increased water temperature (p = 0.011) and increased with the number of filters needed to obtain 1500 ml of filtered water (p = 0.016). Thus the OR for a positive observation increased by a factor of 2.2 per 3 °C decrease in temperature, and with factor of 2.1 for each additional filter.

## Discussion

We directly compared an eDNA approach to traditional methods for monitoring endangered amphibian species in an area with a large number of suitable pool frog habitats (area of approx. 7 km^2^) in proximity to a planned large construction site. Despite high background of DNA inhibitors (humic substances) in the pools, high pH and shifting pool water temperatures during the season, we successfully detected *P. lessonae* DNA. Based on the occupancy analysis we show that the overall detection probability of eDNA was similar to the detection probability of traditional techniques. Still, this resulted in a higher prediction of occupied pools when using eDNA compared to traditional methods.

Statistical analysis reinforced intuitive interpretations that detection of *P. lessonae* depended on the method and time period. The eDNA method tended to detect fewer frogs in June (p = 0.06), but had more detections in May, July, and especially in August (p < 0.001) as compared to traditional observations. These results are reasonable, as the traditional observations are supposed to have their highest observation rates during the peak spawning period in June, when the characteristic loud mating call make the frogs easily detectable.

Still, a concern with eDNA detection is the risk of obtaining false-positive detections as the result of sampling or laboratory contamination^24,25^ and unspecific amplification^26^. In the case of endangered species, a positive detection of the target organism can initiate a costly chain of events in an attempt to conserve the organism. To avoid unnecessary response actions, it is highly desirable to be able to determine whether a positive result is a true detection. Our positive controls for environmental DNA detection were tissue-derived DNA that may cause cross-contamination. To avoid or minimize the misinterpretation of potential sampling and laboratory contaminants as false-positive results, we ran multiple negative controls, such as trip blanks (four winter samples), site blanks (a water body from which *P. lessonae* is absent), and method blanks (filter blanks, extraction blanks and qPCR blanks). A second source of false positives is unspecific amplification of non-targets. We analyzed melting curves for all samples and checked for unspecific amplification by comparing sample plots with single peaked positive controls. Still, metabarcoding (sequencing) of marker genes providing species resolution of amphibian represents a logical next step to validate for false positives, such us unspecific amplification of none *P. lessonae* DNA.

Imperfect detection, false negatives due to the rarity of potential targets, such as eDNA or calling males and silent individuals, can be modeled. In the case of eDNA approaches, multiple covariates can potentially influence DNA recovery and detection of *P. lessonae* and thus hamper reliably estimates of occupancy, abundance and biomass^5,26–28^. Our models predict that variations in observation rate between the methods could be partially explained by the time period (lifecycle stage). At the same time it is unclear how the behavior of the reproducing individuals change during the season. It could be expected that a peak of activity would occur during the spawning period in May to mid-June, further enhanced by release of eDNA during oviposition, but also potential peaks after hatching and subsequent foraging by juveniles. An increase in targeted eDNA concentration has been observed in other water bodies due to the release of gamete and oviposition during the spawning period as shown for the endangered Macquarie perch^29^ and eastern hellbender^30^.

Interestingly most false negatives based on the eDNA approach are found during the spawning period and this could either be a consequence of lower abundances or more restricted spatial movements within the ponds (e.g. territorial behavior) or both, which would suggest adaptions in the sampling strategy of individual ponds. The lifecycle dependent observation rate may also be one explanation to the weak association between observed individuals and DNA concentration, which need further attention if this method is to be considered as a standalone tool for traditional field estimates in the future. Furthermore, lifecycle dependent observation rates may merit for the potential of eDNA approaches in discriminating sites inhabited by different life cycle stages after additional experiments, such as using the ratio between mitochondrial and nuclear derived eDNA^29^.

In earlier studies, humic substances and increased temperature were previously found to decrease eDNA detection by hastening degradation and shortening DNA persistence time in marine systems^12,29^ and these factors might be another source of false negatives. The environmental matrix (i.e. humic substances) seemed to play a minor role in PCR inhibition as shown by an average of 1.7% inhibition of tadpole DNA amplification in the natural matrix when compared to molecular grade water. Interestingly, the highest concentration of DOC is generally found in August to September (between 30 to 50 mgL^−1 ^^31^,) explaining the increased number of filters used during extraction of DNA in the later part of the season, also coinciding with the highest eDNA observation probabilities. High DOC concentrations may reduce eDNA break down by light and ultraviolet radiation^26,27^. On the other hand, the lower pond temperature in August, in relation to July, correlated with a higher observational rate of eDNA, as previously observed in other studies (e.g.^32^).

To conclude, we argue that reliable estimates of observation rates using eDNA can be obtained by accounting for life history and environmental variables that influence detection, such as lifecyle stage and temperature. For example, presence of small foraging frogs in late season correlated to higher observation rates based on eDNA. How the activity and tissue composition of the different lifecycle stages and environmental conditions determine DNA recovery and stability needs to be further investigated.

Thus the application of eDNA approaches warrants careful consideration of field design, choice of molecular reagents and analytical approach. By sampling single pools at multiple sites and during various occasion, using a DNA extraction kit optimized for a high background of humic substances and a novel qPCR protocol, we successfully detected an endangered aquatic species across a variety of pools differing in water chemistry. However, the emergence of eDNA as a superior tool in large-scale monitoring programs for invasive or endangered species deserves particular attention to environmental influence on imperfect detection and quantification, population dynamics and physiological conditions of the target species.

## Materials and Methods

### The sampling site

The study area is located at the Swedish East coast (Baltic Sea) in the eastern part of the County Uppland close to Forsmark (Lat 60° 22′ N, Long 18° 11 E, see Fig. 1). In this flat area of land up-lift and typically lime-rich soils, a succession from shallow sea bays to ponds and wetlands with a high pH (7–9)^33^ and a characteristic species composition occurs. In these habitats a number of endangered species are found, such as the pool frog (*Pelophylax lessonae*), fen orchid (*Liparis loeselii*), Geyers whorl snail (*Vertigo geyeri*) and flea sedge (*Carex pulicaris*). The area east of Forsmark contains a part of the total habitat of pool frogs in Sweden. The Swedish nuclear fuel waste and management company (SKB) is in an ongoing licensing process to construct a geological repository for spent nuclear fuel in the area. The construction phase will involve redirection of groundwater, which could potentially affect some wetlands in the area. Consequently, an extensive monitoring program has been launched in order to describe nature values and to be able to surveil these during the planned construction phase e.g. threatened species, vegetation, groundwater levels and water chemistry^34,35^.

### The study species

The pool frog (*Pelophylax lessonae*) is a green brown, about 50–90 mm large frog, very similar to the related marsh frog (*P. ridibundus*) from which it can be distinguished by its greater midfoot tuber. Pool frogs occur all over Europe and further east of the Ural Mountains. They are missing from the Iberian Peninsula (except some local introductions), Italy (except the northern part) and most of Scandinavia (http://www.iucnredlist.org/details/58643/0). In Scandinavia *P. lessonae* occurs in about 100 permanent ponds or small lakes, two regions located in southern Norway and along the coast of Northern Uppland in Sweden. Sixty local extinctions have been recorded from 1962 to 2001. During a Swedish survey in 2001, reproduction occurred in 69 ponds while 30 spawning sites were found without reproduction. In 1989 their total population size was estimated to 5,000–6,000 adult animals and 2001 to about 10,000 in Sweden (for details see http://artfakta.artdatabanken.se/taxon/100119).

### Sampling scheme

In total seventeen ponds in the area were visited at least twice during spring and summer 2016 to collect water samples for eDNA analysis (see Fig. 1 detailing sampling sites). The sampling campaign was coordinated with ongoing field studies of *P. lessonae*. Thus four ponds, which were part of a daily monitoring program (see below), were sampled more intensively for eDNA (i.e. at six occasions), including three exclusive visits during May. The remaining pools were visited two or three times throughout the period from July to August, and the timing of the sampling was chosen to match the on-going monitoring programs in the area (see below). Thus in total 60 water samples were included in this study, and 49 of these were matched with observations from traditional methods. These samples were grouped into four time periods, roughly corresponding to the lifecycle of the pool frog; May (early spawning season), June (peak spawning season), July (post spawning) and August (small frogs). In addition, water samples from four ponds were collected in early spring as negative controls (i.e. trip blanks).

### Water temperature

Submersed data loggers, so called Mini-Divers, made hourly temperature measurements in 9 of the 17 ponds at a depth of 5 cm sheltered from direct sunlight.

### Traditional monitoring

Seventeen ponds were visited in June as a part of SKB’s regular monitoring program that has been running since 2012^35^. In short, ponds were visited twice during the mating period and for one hour at each occasion. All calling males and silent observed individuals were counted. The activity of *P. lessonae* during the spawning period is highly dependent on the water temperature. Reproduction does not start before the water has reached a temperature of at least 16 °C^21^, and 19 °C is needed for a successful completion of the larval cycle^36^. In addition to the regular program, six ponds were visited to estimate presence of spawn in July, and fourteen ponds were visited at the end of the summer to count small and adult frogs. In May 2016, SKB launched a six weeks intensive monitoring study of ponds with known occurrences of *P. lessonae*^37^. In that study each pond was visited daily, and calling and sighted individuals following the same procedures as in the regular monitoring program.

### eDNA sampling

Water samples for each pond were taken from at least three sites, generally at different shores of the pond. The water was taken with a telescopic grabber pole using 0.5 L glass bottles that were decontaminated with bleach. The three samples were pooled and kept cooled in the dark until further processing within 6 hours. Using a peristaltic pump water was filtered through 0.45 µm sterivex filters with filtered volumes ranging from 200 to 1500 mL filter^−1^ at a speed ranging between 6-12 min 100 mL^−1^ depending on the number of filters used. Filtering equipment was decontaminated with bleach prior to usage. Filters were stored at −20 °C until DNA extraction. DNA was extracted using MoBio Powersoil DNA extraction kit following the manufacturer’s instruction (Qiagen, Carlsbad, CA, USA). DNA was quantified using Quant-iT™ PicoGreen® dsDNA Reagent (Thermo Fisher Scientific) and a Polarstar Omega Microplate reader (BMG labtech, Ortenberg, Germany).

### qPCR

A new PCR protocol was developed for the pool frog (*Pelophylax lessonae*). We designed a set of species-specific primers for *P. lessonae* targeting a small region of the mitochondrial DNA (mtDNA) cytochrome b (database entry Q94RW7_PELLE) and cytochrome c1 (database entry G8HSZ6_PELLE). Primers were designed using PRIMER3 with default settings, with the exception of the product size range which was set to 100–250 bp. Resulting primers were blasted against ncbi nt/nr database to evaluate specificity for *P. lessonae*. Primer pairs that returned non-target sequences of any other species were discarded. This resulted in five primer pairs, as given in Table 1, that were subject to further evaluation for qPCR. For assay evaluation, we used positive controls representing DNA from a juvenile *P. lessonae*, an environmental sample spiked with tadpole *P. lessonae* DNA and a negative control (a sample from large lake Bolundsfjärden close to the Baltic Sea just outside the sampling area). The DNA from the tadpole was also diluted to a four-fold dilution series (0.27 ng/µl to 0.27 pg/µl). Based on the outcome of the evaluation experiments (i.e. linearity of the standard series, homogeneity of melting curves and limit of detection) the final qPCR protocol was as following:

Each PCR reaction mixture (20 μL) contained 3 μL of DNA solution with 250 nM forward and reverse primers (cytC1_676_F, 5°-GACCCCGTTCTCTACCAACA-3°; cytC1_850_R, 5°-ATCCCAGAAGGCCGATAGAT-3°) in a 1 × PCR master mix (Quant−IT picogreen dsDNA kit, Bio-Rad, Hercules, CA, USA). PCR reactions were performed under thermal cycler conditions of 30s at 95 °C, and 40 cycles of 5s at 95 °C and 30s at 60 °C on a CFX connect real time system (Bio-Rad).

Three wells were used as a none-template negative control for all qPCR assays; an amplification signal was not observed in these wells and the blank samples. Specificity of the qPCR method was monitored with melting curves. This revealed unspecific amplification in samples where amplification products could be observed first after 38 cycles.

Four-fold dilution series, 0.81 ng to 0.81 pg per 20 μL PCR reaction mixture, were amplified in duplicates in all qPCR assays to produce standard curves for quantification. The serial dilution was made from a single extracted *P. lessonae* tadpole that was purified using the MoBio Powersoil DNA extraction kit. The R-squares of the standard curve for the qPCR experiments ranged from 0.985 to 0.998. While quantitative values for each sample were estimated from averages of three qPCR runs, presence was defined by a single positive in an individual qPCR run (see supplementary material for raw data). In addition we tested qPCR inhibition by the natural sampling matrix. We spiked three natural samples with a four-fold dilution series of *P. lessonae* tadpole DNA and compared the resulting Cq values with results from dilution series in milliQ.

### Statistical analyses

Presence data rises from a two-part process: The species occurs in the area of interest and the species is discovered by the investigator. Thus non-detection of a species at a site does not imply that the species is absent. MacKenzie *et al*.^38^ and Tyre *et al*.^39^ introduced a statistical method that makes it possible to tease out the detection probabilities from presence data. We used this site occupancy analysis to estimate the detection probabilities for observations based on the traditional method, and the eDNA method, respectively (R-package “unmarked”, using the model fitting function “occu” https://cran.r-project.org/web/packages/unmarked/vignettes/unmarked.pdf) and occupancy probability of the pools. Presence/absent data was analyzed with time period as a covariate in the occupancy model.

In addition, we evaluated the agreement between the eDNA and the traditional method by a simple likelihood ratio test (prescence/absence) and by describing the number of frogs as a function of the measured eDNA concentration. The latter test was done with a poisson regression (implemented as a general linearized model and accounting for over dispersion). Next we examined to what extent the probabilities of observing *P. lessonae* by the eDNA method varied with season, and contrasted the method against the traditional method across the seasons. For this purpose, the presence/absence data from the two methods were analyzed jointly in a logistic regression. The model was implemented as a general linearized model (with a logit link function and a binomial error distribution) and it included the factors method (eDNA vs traditional), season (May, June, July, August) and the interaction between method and season. To account for the differences in frog population between sites, pond (17 levels) was included as a blocking factor in the analysis. Finally, we related the eDNA observation rate to variation in environmental conditions between sampling occasions (within pond), with a logistic regression, using pond as a blocking factor in the analysis. All generalized linear models were implemented in JMP® (version 13.1.0, SAS institute Inc., Cary, NC) using the Firth adjustment maximum likelihood method.

## Electronic supplementary material

Dataset 1
